# Supplementary material for: Ooctonus vulgatus (Hymenoptera, Mymaridae), a potential biocontrol agent to reduce populations of Philaenus spumarius (Hemiptera, Aphrophoridae) the main vector of Xylella fastidiosa in Europe
Source: PeerJ. 2020 Mar 24;8:e8591. doi: 10.7717/peerj.8591 (PMC7100589; doi:10.7717/peerj.8591)

**Supplementary data**

**Appendix S1:** **Illustration of the hatching experiment to quantify parasitism rates in four sampling sites.**

Bunches of branches of *Cistus monspeliensis* were collected on four sites, at the following GPS coordinates:

- 42.984205 °N, 9.395287 °E (Ersa);
- 42.338849 °N, 9.180636 °E (Tralonca);
- 42.274756 °N, 9.487185 °E (Canale-di-Verde);
- 41.931726 °N, 9.343731 °E (Ventiseri).

After inspection of the back of each leaf of *C. monspeliensis* collected in the Corsican maquis, parts of leaf on which egg series were observed were sampled and put on moistened filter paper in Petri dishes (A). Panel B shows a zoom on the leaf, the white arrow pointing to the *P. spumarius* egg cluster. Leaf cuttings were observed under a binocular microscope to confirm the presence of *P. spumarius* eggs. Panels C, D and E show several groups of *P. spumarius* eggs. The orange pigmented spots and the black shields that can both be seen especially in panels C and D are respectively the future eyes of the nymph and the “egg burster” used by the nymph to break the egg shell (Weaver and King 1954). Panel E shows an egg cluster embedded in the frothy cement described by Weaver and King (1954). In panel D, the egg on the right has already hatched whereas the egg on the left hasn’t.

Finally, panel F shows a first instar nymph of *P. spumarius* in dorsal view while panel G show a male of *O. vulgatus* in ventral view.

Photo credit : Xavier Mesmin INRA.

**References**

Weaver CR, King DR. 1954. Meadow spittlebug. *Research bulletin of the Ohio Agricultural Experiment Station* 741:1–100.


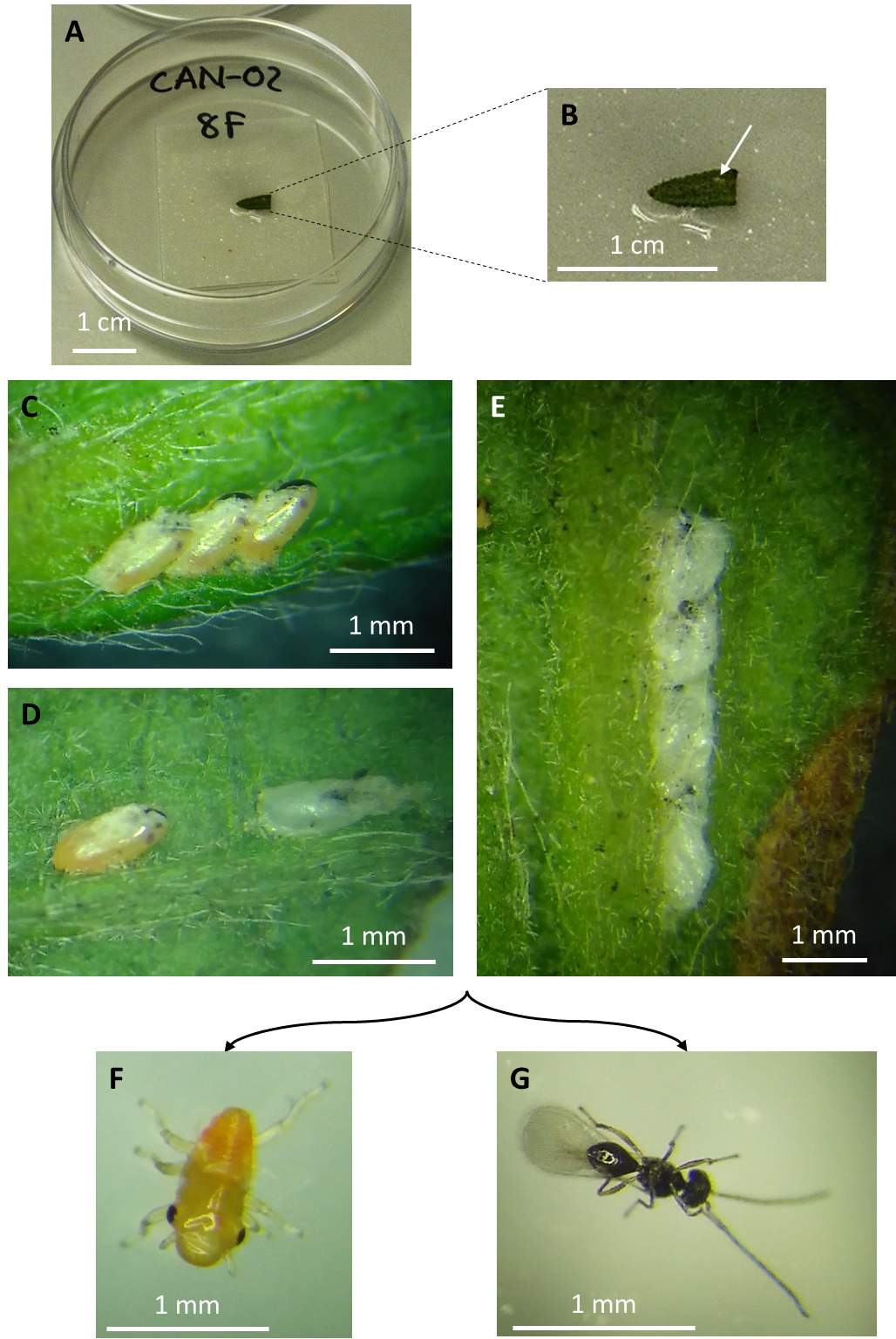

Supplement: Appendix S1 — Bunches of branches of Cistus monspeliensis were collected on four sites, at the following GPS coordinates: 42.984205°N, 9.395287°E (Ersa); 42.338849°N, 9.180636°E (Tralonca); 42.274756°N, 9.487185°E (Canale-di-Verde); 41.931726°N, 9.343731°E (Ventiseri). After inspection of the back of each leaf of C. monspeliensis collected in the Corsican maquis, parts of leaf containing whitish clusters were cut and put on moistened filter paper in Petri dishes (A). (B) shows a zoom on the leaf, the white arrow pointing to the P. spumarius egg cluster. Leaf cuttings were observed under a binocular microscope to confirm the presence of P. spumarius eggs. (C–E) show several groups of P. spumarius eggs. The orange pigmented spots and the black shields that can both be seen especially in (C) and (D) are respectively the future eyes of the larva and the characteristic “egg burster” used by the larva to break the egg shell (Weaver & King, 1954). Panel E shows an egg cluster embedded in the frothy cement described by Weaver & King (1954). In panel D, the egg on the right has already hatched whereas the egg on the left hasn’t. Finally, panel F shows a first instar larva of P. spumarius in dorsal view while panel G show a male of O. vulgatus in ventral view. [file peerj-08-8591-s001.docx]
