# Supplementary material for: Ooctonus vulgatus (Hymenoptera, Mymaridae), a potential biocontrol agent to reduce populations of Philaenus spumarius (Hemiptera, Aphrophoridae) the main vector of Xylella fastidiosa in Europe
Source: PeerJ. 2020 Mar 24;8:e8591. doi: 10.7717/peerj.8591 (PMC7100589; doi:10.7717/peerj.8591)
Supplement: Appendix S2 — This appendix provides details regarding the species distribution modelling framework used in the study [file peerj-08-8591-s002.docx]

**Appendix S2. Supplementary methods**

This appendix contains details regarding the species distribution modelling framework used in the study.

Occurrences of *O. vulgatus* mined in the literature and the GBIF database (GBIF.org 2019) (Tables S1&S2). Valid data were used as input data for species distribution modelling. We used species distribution models (SDMs), which are correlative approaches, to survey the potential distribution of *O. vulgatus*. Such models aim at establishing a statistical relationship between a presence-absence dataset for a given species and a set of selected environmental descriptors (Peterson et al. 2011), usually climate proxies. The resulting models can be used to assess species distribution or the impact of climate change on their range (Guisan et al. 2017). It must be noted that SDMs depict the realized niche of a species (Hutchinson 1957) hence they can fail to fully capture the environmental range (Broennimann et al. 2007). However, SDMs are considered to usually perform well in depicting species distribution and assessing the effects of global changes (Araújo et al. 2005; Elith et al. 2010; Godefroid et al. 2016; Hirzel et al. 2006; Peterson 2003).

The models were based on the bioclimatic descriptors available from the Worldclim 2.0 database (Fick & Hijmans 2017). Data come as a raster with a resolution of 2.5-minute spatial resolution which corresponds to about 4.5 km at the equator. We used a set of eight climate descriptors representative of both temperature and precipitation constraints: mean temperature of wettest quarter (referred to as bio8 in the Worldclim 2.0 database), mean temperature of driest quarter (bio9), mean temperature of warmest quarter (bio10), mean temperature of coldest quarter (bio11), precipitation seasonality (bio15), precipitation of wettest quarter (bio16), precipitation of driest quarter (bio17), precipitation of warmest quarter (bio18) and precipitation of coldest quarter (bio19) (Hijmans et al. 2005). These bioclimatic variables are derived from monthly temperature and rainfall averages between 1970 and 2000 (Fick & Hijmans 2017). Although this is a key step as it can have strong impact upon the resulting model outputs (Peterson & Nakazawa 2008), choosing appropriate climate descriptors is difficult. For that reason, and in absence of formal knowledge about climatic factors constraining the distribution, constituting several sets of predictors and performing modelling with each of them is recommended (Godefroid et al. 2019; Qiao et al. 2015). Resulting models that show good performance are afterwards used to build a consensus model (see details below). We adopted that strategy and calibrated the models using three groups of bioclimatic variables. The first group denoted as CLIM1 comprised bio8, bio9, bio10 and bio11. It thus emphasized the impact of temperature constraints upon *O. vulgatus* distribution. We defined a second dataset (CLIM2) by adding the variable bio15 to CLIM1 to highlight the precipitation constraint. Finally, we built a third dataset (CLIM3) assembling CLIM1 and the bioclimatic variable bio16, bio17, bio18 and bio19 to fully account for both extreme temperatures and precipitations in the models.

We used the Maxent algorithm that relies on the maximum entropy method (Phillips et al. 2006). Maxent considers environmental conditions prevailing at locations where the target species is present but also environmental conditions encountered in a set of locations where no records exist for the species (background locations: (Elith et al. 2011). Background points are generally randomly distributed within an area whose extent and location has marked impact upon the model performances (VanDerWal et al. 2009). A total of 10,000 random background points were generated within areas corresponding to North America (longitudes -140°W and -30°E and latitudes 27°N and 54°N) and Europe (longitudes -20°W and 40°E and latitudes 35°N and 63°N) where valid occurrences of *O. vulgatus* were available.

The parametrization of Maxent involves choosing between different non-linear data transformations (referred to as feature classes or FCs) that allow rendering the complex species’ response to environmental constraints (Elith et al. 2006). Feature classes encompass linear, quadratic, product, hinge and threshold (see Phillips & Dudik 2008, for details). Maxent also involves a regularization multiplier (RM) whose purpose is to reduce overfitting (Merow et al. 2013). Although Maxent come with a set of default parameter values, they might not be optimal in all situations (Shcheglovitova & Anderson 2013) and a sensible approach to model parametrization is to search for optimal values of these parameters given the available dataset (Radosavljevic et al. 2014). For that purpose, we fitted 48 Maxent models using 6 RM combinations (L, LQ, H, LQH, LQHP, LQHPT with L=linear, Q=quadratic, H=hinge, P=product and T=threshold) and FC values (8 values ranging from 0.5 to 4 with increments of 0.5). This initial optimization procedure was performed using the R language (R core team 2019) and the R package ENMeval (Muscarella et al. 2014). The optimal combination of FC and RM values corresponded to the model giving the minimum AICc values (see Muscarella et al. 2014, for detailed explanations).

Optimal FC and RM combinations were determined for each of our three bioclimatic datasets (CLIM1, CLIM2 and CLIM3). The resulting parameters were then used to fit a set of ten replicate Maxent models using 10,000 background points and a training dataset constituted by a random subset of 70% of the occurrences. A total of 30 models was thus fitted. The performance of each model was evaluated using the remaining 30% of available occurrences using two metrics: the area under the receiver–operator curve (AUC, Fielding & Bell 1997) and the true skill statistics (TSS, Allouche et al. 2006). This procedure allowed us to select the best combination of Maxent and bioclimatic datasets and to discard poorly performing models. Models associated to AUC < 0.8 were excluded from further analyses (Vicente et al. 2013). Maxent produces habitat suitability maps (logistic output ranging from 0 to 1) that were transformed into binary projections using the threshold that optimized the TSS statistics on the testing data (Guisan et al. 2017). Maxent replicate models were fitted and evaluated using the R package biomod2 (Thuiller et al. 2009).

Two different outputs were generated using the set of model prediction. i) Binary predictions were averaged to produce the committee (consensus) averaging (Araújo & New 2007; Marmion et al. 2009) showing the likelihood of the presence of *O. vulgatus*. This consensus model ranges from 0 (all the models predict absence) to 100 (all the models predict presence) and ii) the median of the logistic outputs (Guisan et al. 2017) of the models that depicts the climate suitability across the different models.

**References**

Allouche O, Tsoar A, and Kadmon R. 2006. Assessing the accuracy of species distribution models: prevalence, kappa and the true skill statistic (TSS). *Journal of applied ecology* 43:1223-1232.

Araújo MB, and New M. 2007. Ensemble forecasting of species distributions. *Trends in ecology & evolution* 22:42-47.

Araújo MB, Pearson RG, Thuiller W, and Erhard M. 2005. Validation of species–climate impact models under climate change. *Global Change Biology* 11:1504-1513.

Broennimann O, Treier UA, Müller‐Schärer H, Thuiller W, Peterson AT, and Guisan A. 2007. Evidence of climatic niche shift during biological invasion. *Ecology letters* 10:701-709.

Elith J, H. Graham C, P. Anderson R, Dudík M, Ferrier S, Guisan A, J. Hijmans R, Huettmann F, R. Leathwick J, Lehmann A, Li J, G. Lohmann L, A. Loiselle B, Manion G, Moritz C, Nakamura M, Nakazawa Y, McC. M. Overton J, Townsend Peterson A, J. Phillips S, Richardson K, Scachetti-Pereira R, E. Schapire R, Soberón J, Williams S, S. Wisz M, and E. Zimmermann N. 2006. Novel methods improve prediction of species’ distributions from occurrence data. *Ecography* 29:129-151. 10.1111/j.2006.0906-7590.04596.x

Elith J, Kearney M, and Phillips S. 2010. The art of modelling range‐shifting species. *Methods in ecology and evolution* 1:330-342.

Elith J, Phillips SJ, Hastie T, Dudík M, Chee YE, and Yates CJ. 2011. A statistical explanation of MaxEnt for ecologists. *Diversity and Distributions* 17:43-57.

Fick SE, and Hijmans RJ. 2017. WorldClim 2: new 1‐km spatial resolution climate surfaces for global land areas. *International journal of climatology* 37:4302-4315.

Fielding AH, and Bell JF. 1997. A review of methods for the assessment of prediction errors in conservation presence/absence models. *Environ Conserv* 24:38-49.

GBIF.org. 2019. GBIF Home Page. Available from: https://www.gbif.org/

Godefroid M, Cruaud A, Streito J-C, Rasplus J-Y, and Rossi J-P. 2019. Xylella fastidiosa: climate suitability of European continent. *Scientific Reports* 9:8844. 10.1038/s41598-019-45365-y

Godefroid M, Rocha S, Santos H, Paiva MR, Burban C, Kerdelhué C, Branco M, Rasplus JY, and Rossi JP. 2016. Climate constrains range expansion of an allochronic population of the pine processionary moth. *Diversity and Distributions* 22:1288-1300.

Guisan A, Thuiller W, and Zimmermann NE. 2017. *Habitat suitability and distribution models: with applications in R*: Cambridge University Press.

Hijmans RJ, Cameron SE, Parra JL, Jones PG, and Jarvis A. 2005. Very high resolution interpolated climate surfaces for global land areas. *International Journal of Climatology: A Journal of the Royal Meteorological Society* 25:1965-1978.

Hirzel AH, Le Lay G, Helfer V, Randin C, and Guisan A. 2006. Evaluating the ability of habitat suitability models to predict species presences. *Ecological modelling* 199:142-152.

Hutchinson GE. 1957. Concluding remarks Cold Spring Harbor Symposia on Quantitative Biology, 22: 415–427.

Marmion M, Parviainen M, Luoto M, Heikkinen RK, and Thuiller W. 2009. Evaluation of consensus methods in predictive species distribution modelling. *Diversity and Distributions* 15:59-69.

Merow C, Smith MJ, and Silander JA. 2013. A practical guide to MaxEnt for modeling species’ distributions: what it does, and why inputs and settings matter. *Ecography* 36:1058-1069. 10.1111/j.1600-0587.2013.07872.x

Muscarella R, Galante PJ, Soley-Guardia M, Boria RA, Kass JM, Uriarte M, Anderson RP, and McPherson J. 2014. ENMeval: An R package for conducting spatially independent evaluations and estimating optimal model complexity forMaxentecological niche models. *Methods in ecology and evolution* 5:1198-1205. 10.1111/2041-210x.12261

Peterson AT. 2003. Predicting the geography of species’ invasions via ecological niche modeling. *The quarterly review of biology* 78:419-433.

Peterson AT, and Nakazawa Y. 2008. Environmental data sets matter in ecological niche modelling: an example with Solenopsis invicta and Solenopsis richteri. *Global ecology and Biogeography* 17:135-144.

Peterson AT, Soberón J, Pearson RG, Anderson RP, Martínez-Meyer E, Nakamura M, and Araújo MB. 2011. *Ecological niches and geographic distributions (MPB-49)*: Princeton University Press.

Phillips SJ, Anderson RP, and Schapire RE. 2006. Maximum entropy modeling of species geographic distributions. *Ecological modelling* 190:231-259.

Phillips SJ, and Dudik M. 2008. Modeling of species distributions with Maxent: new extensions and a comprehensive evaluation. *Ecography* 31:161--175.

Qiao H, Soberón J, and Peterson AT. 2015. No silver bullets in correlative ecological niche modelling: insights from testing among many potential algorithms for niche estimation. *Methods in ecology and evolution* 6:1126-1136.

Radosavljevic A, Anderson RP, and Araújo M. 2014. Making better Maxentmodels of species distributions: complexity, overfitting and evaluation. *Journal of Biogeography* 41:629-643. 10.1111/jbi.12227

Shcheglovitova M, and Anderson RP. 2013. Estimating optimal complexity for ecological niche models: a jackknife approach for species with small sample sizes. *Ecological modelling* 269:9-17.

R Core Team 2019. R: A language and environment for statistical computing. R Foundation for Statistical Computing, Vienna, Austria. URL https://www.R-project.org.

Thuiller W, Lafourcade B, Engler R, and Araújo MB. 2009. BIOMOD–a platform for ensemble forecasting of species distributions. *Ecography* 32:369-373.

VanDerWal J, Shoo LP, Graham C, and Williams SE. 2009. Selecting pseudo-absence data for presence-only distribution modeling: how far should you stray from what you know? *Ecological modelling* 220:589-594.

Vicente JR, Fernandes RF, Randin CF, Broennimann O, Gonçalves J, Marcos B, Pôças I, Alves P, Guisan A, and Honrado JP. 2013. Will climate change drive alien invasive plants into areas of high protection value? An improved model-based regional assessment to prioritise the management of invasions. *Journal of environmental management* 131:185-195.
