# Supplementary material for: Ooctonus vulgatus (Hymenoptera, Mymaridae), a potential biocontrol agent to reduce populations of Philaenus spumarius (Hemiptera, Aphrophoridae) the main vector of Xylella fastidiosa in Europe
Source: PeerJ. 2020 Mar 24;8:e8591. doi: 10.7717/peerj.8591 (PMC7100589; doi:10.7717/peerj.8591)

Figure S1: Maximum likelihood phylogenetic tree including the newly generated sequences and all *COI* barcodes available in BOLD for *Ooctonus* species (last access July 12,2019).

Bootstrap (100 replicates) at nodes. The newly generated sequences are in red. The only four sequences identified as *O. vulgatus* in BOLD are in blue.

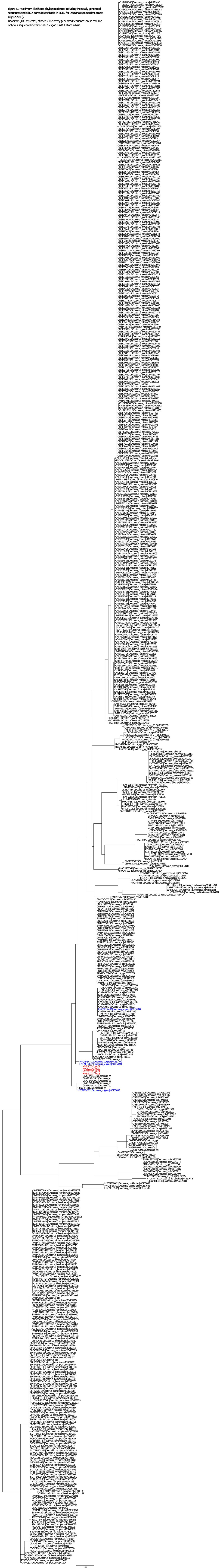

Supplement: Figure S1 — Bootstrap (100 replicates) at nodes. The newly generated sequences are in red. The only four sequences identified as O. vulgatus in BOLD are in blue. [file peerj-08-8591-s003.pdf]
